# Supplementary material for: Effectiveness and Safety of Ayurvedic Medicines in Type 2 Diabetes Mellitus Management: A Systematic Review and Meta-Analysis
Source: Front Pharmacol. 2022 Jun 8;13:821810. doi: 10.3389/fphar.2022.821810 (PMC9213670; doi:10.3389/fphar.2022.821810)
Supplement: Supplementary file 1 [file DataSheet1.zip › Supplementary Material/Forest and Funnel Plots/Trigonella foenum-graecum (versus OAD)/TC.pdf]

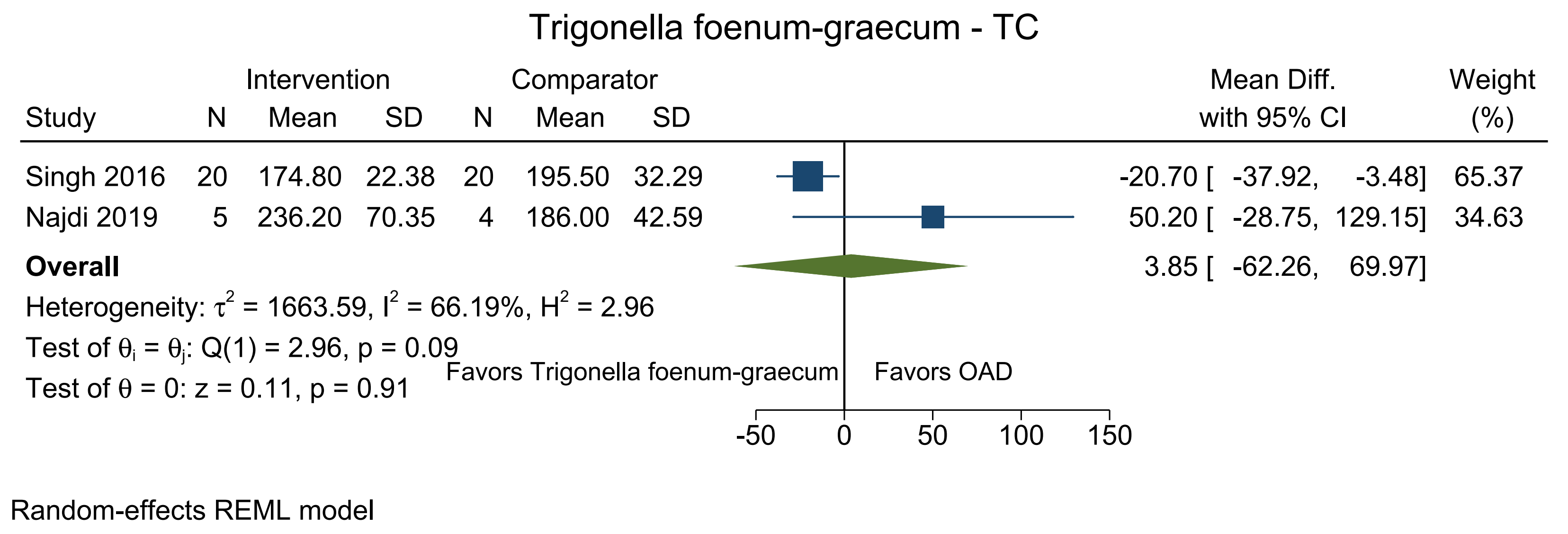
Trigonella foenum-graecum - TC
Intervention
Comparator
Study
N
Mean
SD
N
Mean
SD
Mean Diff. with 95% CI
Weight (%)
Singh 2016 20 174.80 22.38 20 195.50 32.29 -20.70 [-37.92, -3.48] 65.37
Najdi 2019 5 236.20 70.35 4 186.00 42.59 50.20 [-28.75, 129.15] 34.63
Overall 3.85 [-62.26, 69.97]
Heterogeneity: τ^2 = 1663.59, I^2 = 66.19%, H^2 = 2.96
Test of $\theta_i = \theta_j$: Q(1) = 2.96, p = 0.09
Test of θ = 0: z = 0.11, p = 0.91
Favors Trigonella foenum-graecum
Favors OAD
-50
0
50
100
150
Random-effects REML model
